# Evaluation of the SPARK Child Mentoring Program: A Social and Emotional Learning Curriculum for Elementary School Students

**DOI:** 10.1007/s10935-021-00642-3

**Published:** 2021-08-17

**Authors:** Amy L. Green, Stephen Ferrante, Timothy L. Boaz, Krista Kutash, Brooke Wheeldon-Reece

**Affiliations:** 1 College of Behavioral and Community Sciences, University of South Florida, 13301 Bruce B. Downs Blvd., Tampa, FL 33612 USA; 2 Group Victory LLC, P.O. Box 39775, Ft. Lauderdale, FL 33339 USA; 3 Department of Mental Health Law and Policy, University of South Florida, 13301 Bruce B. Downs Blvd., Tampa, FL 33612 USA; 4 Department of Child and Family Studies, University of South Florida, 13301 Bruce B. Downs Blvd., Tampa, FL 33612 USA; 5 The SPARK Initiative, Inc, 913 S. Parsons Ave. Suite C, Brandon, FL 33511 USA

**Keywords:** Social and Emotional Learning, Resilience, Elementary school

## Abstract

Social and Emotional Learning (SEL) programs seek to enhance social and emotional competencies in children, including self-awareness, self-management, social awareness, relationship skills, and responsible decision-making. By means of direct instruction regarding social and emotional competencies, SEL programs have the potential to strengthen resilience in children and thus their capacity to effectively cope with life’s challenges. Strengthening resilience in children who are repeatedly exposed to adverse experiences, particularly those from economically disadvantaged minority backgrounds, is of particular importance and has implications for the prevention of a multitude of problems later in life. Our study reports the result of an investigation of the SPARK Child Mentoring program, a resilience-focused SEL program designed to reduce risk factors, uncover innate resilience, promote natural emotional well-being, and facilitate school success. We employed a randomized controlled trial comprising 94 elementary school students that included pre- and post-intervention measurements. After controlling for pre-intervention levels, we found a significant difference between students’ understanding of underlying program principles; communication, decision making, and problem-solving skills; emotional regulation; and resilience for students who received the intervention compared to students who did not receive the intervention. These results provide initial evidence for the efficacy of the SPARK Child Mentoring program with a diverse sample of elementary school students and adds to the existing literature base concerning positive outcomes associated with SEL programs. We discuss implications for future research focused on long-term preventive effects of the program and the characteristics of students most likely to benefit from it.

## Introduction

Decades of research highlight the important role of social and emotional competencies in child development. Social and emotional competencies facilitate a child’s success in developing and maintaining healthy relationships, coping with difficulties, and maintaining overall health and well-being. Children who lack core social and emotional competencies experience greater risks for future academic, behavioral, and social impairments (Thayer et al., 2019). As a result, schools often implement Social and Emotional Learning (SEL) programs to directly teach social and emotional competencies in the classroom setting. A strong evidence base demonstrates the important benefits of SEL programs, including positive impacts on social-emotional skills, mental health, academic functioning, overall health and well-being, and risk-taking behaviors (Corcoran et al., 2018; Dowling et al., 2019; Durlak et al., 2011; Taylor et al., 2017). From a prevention standpoint, the elementary school years represent an important stage of development for directly addressing social and emotional competencies. Doing so is especially important for children from social and economic backgrounds who have traditionally faced multiple and ongoing hardships. A risk to these children is that these hardships will interrupt their developmental trajectory and lead to further problems later in life. As demonstrated by previous research, the knowledge and skills promoted within SEL interventions are associated with positive developmental trajectories (Taylor et al., 2017). While resilience is often considered in the context of adaptation related to hardship, it is also an important aspect of overall positive development (Masten, 2014).

### Social and Emotional Learning

Social and Emotional Learning (SEL) is the process through which children develop knowledge, attitudes, and skills for understanding and managing emotions, setting and achieving goals, showing empathy, maintaining positive relationships, and making responsible decisions (CASEL, 2020). Social and emotional competencies are key to children developing into healthy and competent young adults and are important for success in school, work, and life (Carroll et al., 2020; Nicoll, 2014). SEL is a process whereby children progress through different developmental tasks such as understanding basic emotional expressions in preschool to understanding unique emotional perspectives in high school. Despite the changing nature of specific tasks associated with SEL, core SEL competencies remain the same throughout development (Denham, 2018).

The Collaborative for Academic, Social, and Emotional Learning (CASEL) has identified five core competencies of SEL: self-awareness, self-management, social awareness, relationship skills, and responsible decision-making. As defined by CASEL, self-awareness refers to the ability to recognize emotions and thoughts and their influence on behavior and assess personal strengths and limitations. Self-management refers to the ability to effectively regulate emotions, thoughts, and behaviors in different situations and to set and work toward goals. Social awareness refers to the ability to take the perspective of and empathize with others, to understand social and ethical norms for behavior, and to recognize available resources and supports. Relationship skills refer to the ability to establish and maintain healthy relationships, communicate well with others, negotiate conflict, and seek and offer help when needed. Finally, responsible decision-making refers to the ability to make constructive choices about personal behavior and social interactions, evaluate consequences of actions, and consider the well-being of self and others (CASEL, 2020).

An important function of the provision of SEL programs in schools is to prevent social-emotional and behavioral problems and promote student well-being and success (Thayer et al., 2019). Carroll and colleagues (2020) point to a growing belief that SEL in educational settings is at least as important as academic content. In fact, the implementation of SEL programs in schools has become a widely accepted component of education. This is evidenced by federal legislation that increasingly supports educating the whole child (Greenberg et al., 2017). Additionally, schools provide an ideal setting where social and emotional competencies can be taught, modeled, practiced, and reinforced.

Many different SEL programs have been developed and implemented in schools across the United States ranging from universal whole-class programs to targeted programs for at-risk children and those with skill deficits (Carroll et al., 2020). Additionally, there has been significant growth in the number of states adopting standards for social and emotional learning and guidance to support implementation in schools (CASEL, 2018). Support for the implementation of SEL programs in schools is further bolstered by teachers, the majority of whom report that SEL should be an important part of a student’s experience in school, particularly in elementary school (Civic Enterprises, 2013).

Growth in the adoption and support of SEL programs is largely due to an increasing evidence base that supports the positive impact of SEL programs on a range of academic, behavioral, emotional, social, and cognitive outcomes for students at all developmental levels (Jones et al., 2017). Two meta-analyses of school-based universal SEL interventions for students in kindergarten through high school provide evidence for positive outcomes of SEL related to improved social and emotional skills, attitudes, behavior, and academic performance (Corcoran et al., 2018; Durlak et al., 2011). There is also evidence for the long-term impact of SEL programs on positive youth development six months to 18 years following intervention on social and emotional assets (i.e., SEL skills and attitudes) and indicators of well-being (i.e., academic performance, emotional distress, and drug use; Taylor et al., 2017). Research also suggests that benefits of SEL in schools often extend beyond positive impacts at the individual student level to overall classroom functioning and school climate (Greenberg et al., 2017; Jones et al., 2017).

When considering the value of SEL programs, it is important to look at improvement in SEL skills as well as prevention of later problems and promotion of resilience (Thayer et al., 2019). From a public health perspective, SEL programs present an opportunity to positively impact the developmental trajectories and well-being of students and potentially prevent or reduce the detrimental effects of adverse experiences (Greenberg et al., 2017). Implementation of programs to promote resilience in the face of adversity is an important strategy for reducing the impact of the accumulation of risk factors on child development (Duch et al., 2019). For children who are more likely to face adverse experiences, such as those from ethnic minority and lower socio-economic backgrounds, developing and strengthening social and emotional competencies through SEL programs is important. In a recent study by West and colleagues (2020), more than 282,000 fourth- through twelfth-grade students from 1033 schools in six districts completed a survey to examine trends in SEL development. Results from this large-scale panel study demonstrated that across all grade levels, economically disadvantaged students reported lower levels of social and emotional competencies (i.e., growth mindset, self-efficacy, self-management, and social awareness) than economically advantaged grade-level peers. African American and Latinx students reported lower levels of self-management and social awareness than their White and Asian grade-level peers. Both African American and Latinx students also reported lower self-efficacy than White students across all grade levels (West et al., 2020). For economically disadvantaged ethnic minority children, promoting resilience by teaching social and emotional skills that foster positive relationships at school, increase academic engagement, and promote social competence and self-efficacy is an important mechanism for the prevention of maladaptive outcomes later in life (Henderson et al., 2016).

### SPARK Child Mentoring Program

The SPARK Child Mentoring program is a resilience-focused school-based SEL program designed to reduce risk factors, uncover innate resilience, promote natural emotional well-being, and facilitate school success. The SPARK Child Mentoring program was developed to meet the needs of elementary students between the ages of eight and ten years. The program covers relevant and relatable topics that cultivate social and emotional skills and help children better understand themselves and others and access their creativity and potential. The topics covered in the program and the nature of the activities used to teach and reinforce the program content are consistent with the SEL skills and developmental tasks unique to this age group. The SPARK Child Mentoring program employs the principles of Mind, Thought, and Consciousness. The principle of Mind represents the energy that powers thought and consciousness and has been conceptualized as the source of inner mental health and wisdom that is available to everyone. Throughout this program, Mind is referred to as the “SPARK.” It is described as the source behind all things in life: everything seen, felt, and experienced. This “SPARK” may also be called “intuition,” “instinct,” or “common sense.” The principle of Consciousness represents awareness and the ability to experience life, and the principle of Thought refers to the ability to think and create a psychological experience from within (Pransky & Kelley, 2014). Based on these principles, a foundational premise of the SPARK Child Mentoring program is that the capacity for positive development and healthy psychological functioning (e.g., resiliency, emotional competency, self-management, self-awareness, self-efficacy, social awareness) is innate and can be drawn-out of all children irrespective of their past socialization or exposure to adverse childhood experiences. By increasing understanding and insights around these principles, children can access and experience their natural, innate well-being and prevent negative developmental outcomes (Kelley, 2003). Uncovering and strengthening these competencies within children can reinforce their resilience and bolster their capacity to manage the relationships, responsibilities, expectations, and challenges they face (Kelley et al., 2017a, 2017b). As suggested by Banerjee et al. (2007), targeting the principles of Mind, Thought, and Consciousness is likely to produce more sustainable change than simply targeting an individual’s thoughts, thought processes, feelings, and behaviors.

Unlike many other SEL programs, the SPARK program does not focus on changing children’s thinking, feelings, or behaviors. Instead, it focuses on helping children realize that when their thinking changes, their experiences, feelings, perceptions, and states of mind also change. Therefore, the goal is to help children realize that when their personal thinking quiets, their mental well-being, common sense, and innate resiliency naturally surface. This differs from other SEL approaches that aim to help children identify and use techniques or strategies to quiet their minds or rid themselves of certain thinking. The SPARK Child Mentoring program is designed to provide students with insights that allow them to notice what they experience when their personal minds quiet and to understand that mental well-being, wisdom, and resilience are always available from within.

### Study Aims

Our overarching goal for this study is to provide a description of an initial evaluation of the SPARK Child Mentoring program. Specifically, we aimed to determine if the SPARK program: (1) increases participants’ knowledge of program content; (2) increases participants’ communication, problem-solving and decision-making skills; (3) increases participants’ emotional regulation skills; and (4) increases participants’ resilience.

## Method

### Setting and Participants

**Fig. 1:**
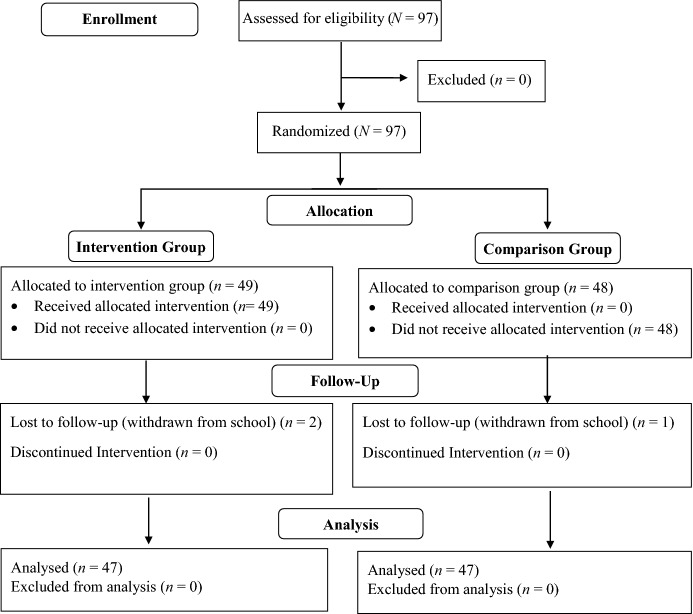
Participant flow chart

For this study, we included 97 fourth- and fifth-grade students from one elementary school located in a large southern school district. The school serves 753 students in pre-kindergarten through 5th grade and is a Title 1 school. The student body is primarily Hispanic (70%) and 92% of students are classified as “economically disadvantaged” based on free or reduced-price lunch status. We obtained written informed consent for participation in the study from parents of 97 students in six classes (four 4th grade classes and two 5th grade classes). We randomly assigned participating classes to either the intervention group or the comparison group. Randomization procedures resulted in three classes being randomized to the intervention group, including one fifth grade class (*n* = 15) and two fourth grade classes (*n* = 16 and *n* = 18), and three classes being randomized to the comparison group, including one fifth grade class (*n* = 15) and two fourth grade classes (*n* = 17 and *n* = 16). Because we selected comparison classes from the same school as intervention classes, there is some potential for contamination, which could lead to rejection of an intervention due to small observed differences between groups (Torgerson, 2001). However, only students in the intervention classes received the program and SPARK facilitators did not interact with students in the comparison classes other than to administer pre- and post-assessments. See Fig. 1 for a participant flow chart.

The final study sample included 94 students with both pre- and post-intervention data. The number of students lost to follow-up from the intervention group (*n* = 2) and the comparison group (*n* = 1) did not differ significantly (Fisher’s Exact Test [2-sided] *p* = 1.00, *N* = 97). Students in the intervention group had a mean age of 9.5 years, were 55.3% male, 17.0% White, and 68.1% Hispanic. The majority of students in the intervention group received free or reduced-price lunch (FRL; 91.5%). Students in the comparison group had a mean age of 9.7 years, were 51.1% male, 21.3% White, and 72.3% Hispanic. Most students in this group received FRL (83.0%). Analyses revealed no statistically significant differences between the intervention and control groups on age (*t*[76.5] = 0.97, *p* = 0.34), gender (*Χ*^*2*^ [1, *N* = 95] = 0.01, *p* = 0.94), race (*Χ*^*2*^ [2, *N* = 49] = 0.37, *p* = 0.83), ethnicity (*Χ*^*2*^ [1, *N* = 97] = 0.14, *p* = 0.71), or whether students received FRL (*Χ*^*2*^ [1, *N* = 94] = 0.02, *p* = 0.89). The degrees of freedom for the test of group differences in age is not a whole number because the Satterthwaite method was used to account for unequal variances.

### Procedure

Program implementation and data collection took place between October 2019 and January 2020. Students in the intervention group completed a pre-intervention questionnaire during the first SPARK session and a post-intervention questionnaire during the final SPARK session. An interval of 15 weeks elapsed between pre- and post-assessment. Students in the comparison group completed the pre- and post- questionnaires on the same days as students in the intervention group.

### Intervention Protocol

The SPARK Child Mentoring program consists of 11 weekly lessons designed to be taught by SPARK facilitators. SPARK facilitators are certified through a comprehensive 4-day professional training program. Facilitators deliver the program using a standardized instruction manual that incorporates group activities, discussions, and games designed to help children understand the content of the program. The model for delivering program content incorporates a variety of mentoring approaches. For example, lessons may be delivered in small or large group settings, one-on-one, by a team of adults working with groups of students, and/or using a peer mentoring model. For our study, a single facilitator delivered the SPARK program to students in the three classes randomly assigned to the intervention condition.

### Fidelity

We used a fidelity monitoring system to monitor the degree to which the SPARK program was delivered as designed. After every session, the facilitator completed a Session Fidelity Rating Scale. This scale contains 23 items that describe the essential components and processes of the intervention. Sample statements include “follow the lesson content,” “knowledgeable of subject matter,” and “promote participants’ potential and resiliency.” The facilitator rates each item on a scale of 1 “Not met” to 4 “Met.” In addition to collecting facilitator self-ratings of session fidelity, we also collected supervisor ratings of fidelity using a Supervisory Fidelity Rating Scale that contains the same 23 items as the Session Fidelity Rating Scale. A program supervisor completed this scale during two random session observations.

The SPARK facilitator completed a Session Fidelity Rating Scale for all 11 sessions of the SPARK program in each classroom. These ratings revealed an average fidelity rating of 3.97 out of 4.00 across the three intervention classrooms. To examine the agreement of fidelity ratings, both the facilitator and supervisor rated two sessions across two different classrooms. Results revealed that the facilitator and the supervisor agreed on fidelity ratings 100% of the time. Based on these fidelity measurement results, which consistently demonstrated high fidelity and compliance ratings, there is evidence that the SPARK facilitator delivered the program with fidelity.

### Attendance

To help ensure that each student received an adequate amount or “dose” of the program, we monitored student attendance of SPARK sessions. Monitoring the “dose” of the intervention received by students helps to indicate that the impact of the program is due to the program content and not external events. At each SPARK session, the SPARK facilitator recorded attendance for each student. While we made every effort to “catch-up” a student who missed a session, recording attendance at each session helped to ensure changes in behaviors and attitudes could be associated with program participation. If a student withdrew from school, the research team marked the student as “withdrawn from school” and excluded the student from analysis. Students across the three intervention classrooms had an average attendance rate of 93%, or roughly 10 out of 11 sessions. This suggests that students received an adequate amount of the intervention to demonstrate program impact.

### Measures

#### Background Information

Parents provided their child’s date of birth, gender, race, and ethnicity on consent forms. The school district provided data on lunch status (i.,e., eligibility to receive free, reduced, or full price lunches) for each student.

#### Level of Knowledge of Program Content

To assess students’ knowledge of program content, we administered the Three Principles Inventory for Youth (3PI; Kelley, 2011). The 11-item 3PI for Youth is a revision of the version used with adults. To ensure this measure could be completed independently by elementary-age students, we simplified the wording of items and the response set. For example, the item “My self-esteem can be affected as a result of people criticizing me or 'putting me down'” was rewritten to read “I don’t feel bad about myself even if people are talking bad about me.” Likewise, the item “If something traumatic happens to me it can damage my mental health” was rewritten to read “If something bad happens to me, it can forever damage my emotions.” The response set was also reduced to a 1 to 5 rating scale ranging from “Strongly Disagree” to “Strongly Agree” from a 1 to 6 rating scale ranging from “Disagree Completely No Exceptions” to “Agree Completely No Exceptions.” The total 3PI score ranges from 11 to 55 with higher scores indicating greater knowledge of program content. The 3PI has demonstrated adequate reliability (Cronbach’s alpha = 0.70) when used with adults (Kelley et al., 2017a, 2017b). Reliability analysis indicated a Cronbach’s alpha of 0.73 for the 3PI in our study sample (using both pre- and post-data).

#### Communication, Decision Making, and Problem-Solving Skills

To assess students’ communication, decision making, and problem-solving skills, the SPARK program staff developed the Communication, Decision Making and Problem-Solving scale (CDP-Child Version). The CDP-Child Version is based on items from the National Life Skills Evaluation System scales, which have demonstrated evidence of reliability and validity (Mincemoyer & Perkins, 2005). The CDP-Child Version includes three items that make up the *Problem-Solving Skills* subscale (e.g., “I look within myself to solve problems successfully” and “If my solution is not working, I will try another solution”), four items that make up the *Decision-Making Skills* subscale (e.g., “I know how to make decisions that are best for me” and “I consider consequences prior to making decisions”), and four items that make up the *Communication Skills* subscale (e.g., “I try to see the other person’s point of view” and “I can communicate my feelings without blaming others”). Item response options range from 1 “Never” to 5 “Almost Always.” The total score on the CDP-Child Version is the sum of all items and ranges from 11 to 55 with higher scores indicating greater communication, decision-making, and problem-solving skills. Subscale scores range from 1 to 5 and are derived by summing items for each subscale and then dividing by the number of items for that subscale. Reliability analysis indicated a Cronbach's alpha of 0.87 for this scale in our study sample (total score—using both pre- and post-data).

#### Difficulties in Emotional Regulation

We used the *Impulse* and *Clarity* subscales from the short form of the Difficulties in Emotional Regulation Scale (DERS-SF; Kaufman et al., 2016) to measure difficulties in emotional regulation. The *Impulse* subscale measures difficulties with impulse control and includes items such as, “When I’m upset, I become out of control” and “When I’m upset, I have difficulty controlling my behavior.” The *Clarity* subscale measures lack of emotional clarity and includes items such as, “I am confused about how I feel” and “I have difficulty making sense out of my feelings.” Each subscale includes 3 items with response options that range from 0 “Almost Never” to 4 “Almost Always.” Subscale scores are derived by summing the items for that subscale and range from 0 to 12. The total score, which ranges from 0 to 24, is obtained by adding the two subscale scores. Lower scores on the DERS-SF indicate fewer difficulties with emotional regulation. The DERS-SF total and subscale scores have demonstrated good internal reliability (0.78 to 0.91) and adequate construct and concurrent validity (Kaufman, et al., 2016). Reliability analysis indicated a Cronbach's alpha of 0.85 for this scale in our study sample (total score—using both pre- and post-data).

#### Resilience

To measure resilience, we used three subscales from the Resiliency Scales for Children and Adolescents (RSCA; Prince-Embury, 2007). The *Sense of Relatedness* subscale includes 24 items and measures students’ perceptions of trust, support, comfort, and tolerance. The *Sense of Mastery* subscale includes 20 items and measures optimism, self-efficacy, and adaptability. The *Optimism* subscale includes 7 items taken from the *Mastery* subscale focuses specifically on students’ beliefs about the future. Response options for items on all three subscales range from 0 “Never” to 4 “Almost Always.” Subscale scores are the sum of the items for each subscale and range from 0 to 96 for the *Sense of Relatedness* subscale, 0 to 80 for the *Sense of Mastery* subscale, and 0 to 28 for the *Optimism* subscale. A total resilience score is calculated by summing the *Relatedness* and *Mastery* subscale scores with higher scores indicating greater resilience. The RSCA scales have demonstrated validity through structural investigations, acceptable internal consistency reliability (0.61–0.94), and test–retest reliability (0.79–0.83; Prince-Embury, 2007, 2011). An analysis of our study sample indicated Cronbach’s alphas of 0.93 and 0.91, respectively, for the *Sense of Mastery* subscale and the *Sense of Relatedness* subscale (using both pre- and post- data).

### Data Analysis

To begin, we compared scores on the pre-intervention questionnaire for the intervention and comparison groups to evaluate the adequacy of random assignment in equating the groups for each condition. Next, we analyzed each of the scales contained within the questionnaire to compare change over time for the intervention group versus the comparison group. We then compared the average pre-intervention scores for the intervention and comparison groups using a one-way analysis of variance (ANOVA). While this would normally be done using an independent groups *t* test, we used a one-way ANOVA for the following two reasons: (1) it yields the same conclusion as the *t* test (*F* = *t*^2^), and (2) since we used ANCOVA to test for differences in change over time, this allows for both tests to be based on the *F* statistic*.* We compared the average post-intervention scores for the two groups using analysis of covariance (ANCOVA). In this latter analysis, we entered the condition variable as a factor in the model and the pre-intervention score for that measure as a covariate (this corrects for bias due to pre-intervention group differences and regression to the mean). From this analysis, we report the test statistic for the condition variable using Type III Sums of Squares (this represents the contribution of the condition variable after adjusting for pre-intervention group differences on the outcome measure). Given clustering of students within classrooms, we used PROC MIXED within SAS v.9.4 for these analyses. We entered the intercept for students clustered within classrooms as a random effect. Twisk and Proper (2004) have argued that this approach is preferable (less biased) than the use of residualized change scores for analyzing change over time in randomized controlled trials. Finally, we present the effect size for that measure using Hedges’ *g*. For this statistic, 0.8 or more indicates a large effect, 0.5 to < 0.8 indicates a medium effect, and 0.2 to < 0.5 indicates a small effect, although these cutoffs are generally not applied rigidly (Cohen, 1992).

## Results

At pre-intervention, the intervention and comparison groups did not differ regarding their level of communication, decision-making, and problem-solving as measured by the CDP-Child Version, in their level of difficulties with emotional regulation as measured by the DERS-SF, nor in their level of resilience as measured by the RSCA. However, the intervention and comparison groups did differ significantly on knowledge of program content (3PI). Students in the comparison group reported higher pre-test ratings of knowledge of program content based on the 3PI compared to students in the intervention group.

### Level of Knowledge of Program Content

Post-intervention scores for the comparison group remained essentially unchanged from the pre-intervention scores on the 3PI. In contrast, for the intervention group, results indicated higher mean post-intervention scores compared to pre-intervention scores. The change from pre to post after controlling for pre-intervention levels was statistically significant for the 3PI. Higher scores on this measure reflected more knowledge of the program content, so the SPARK group significantly increased their knowledge compared to the comparison group (see Table 1). The Hedges' *g* effect size for the difference between pre- and post-intervention 3PI scores was 1.06, which is a large effect size.

**Table 1:** Analysis of Variance (ANOVA) of pre-intervention scores and of post-intervention scores for students in the intervention group (*n* = 47) compared to students in the comparison group (*n* = 47)

	Means			
	Intervention group	Comparison group	*F*^a^	*P*
Knowledge of three principles (3PI)				
Pre-intervention	36.66	39.45	4.61	.035
Post-intervention	45.51	39.00	26.86	< .0001
Total CDP score				
Pre-intervention	35.77	37.98	0.63	.430
Post-intervention	44.60	39.15	15.24	.0002
Communication skills subscale				
Pre-intervention	2.94	3.26	1.99	.162
Post-intervention	3.86	3.35	8.13	.005
Decision-making skills subscale				
Pre-intervention	3.23	3.44	0.55	.461
Post-intervention	4.09	3.62	10.90	.001
Problem-solving skills subscale				
Pre-intervention	3.70	3.74	0.03	.871
Post-intervention	4.27	3.77	12.97	.0005
Total DERS-SF score				
Pre-intervention	9.68	9.77	0.00	.946
Post-intervention	4.00	10.11	40.96	< .0001
Clarity subscale				
Pre-intervention	4.02	4.62	0.73	.396
Post-intervention	2.38	4.38	13.42	.0004
Impulse subscale				
Pre-intervention	5.66	5.15	0.39	.536
Post-intervention	1.62	5.72	53.99	< .0001
Total resilience score				
Pre-intervention	107.06	117.06	1.42	.237
Post-intervention	132.23	113.13	13.53	.0004
Relatedness subscale				
Pre-intervention	57.26	64.53	2.67	.106
Post-intervention	71.43	61.34	11.07	.001
Mastery subscale				
Pre-intervention	49.81	52.53	0.47	.495
Post-intervention	60.81	51.79	14.67	.0002
Optimism subscale				
Pre-Intervention	17.30	18.17	0.58	.450
Post-intervention	21.11	17.45	15.79	.0001

^a^PROC MIXED (SAS v. 9.4) was used to adjust for clustering of students within classrooms. The *F* for pre-intervention is the condition effect at pre-intervention (*df* = 1, 88), and the *F* for post-intervention is the test of the condition effect after covarying out the pre-intervention effect (*df* = 1, 87)

3PI = Three Principles Inventory; CDP = Communication, Decision-Making, and Problem-Solving Scale (Child Version); DERS-SF = Difficulties in Emotional Regulation Scale–Short Form

### Communication, Decision Making, and Problem-Solving Skills

On the total CDP scale and the three CDP subscales, the post-intervention scores for the comparison group remained essentially unchanged compared to the pre-intervention scores. For the intervention group, results indicated higher mean post-intervention scores compared to pre-intervention scores. Comparison of the intervention and comparison groups on change from pre to post after controlling for pre-intervention levels was statistically significant for the total CDP scale, as well as the *Communication Skills* subscale, the *Decision-Making Skills* subscale, and the *Problem-Solving Skills* subscale. Higher scores on these scales reflect more skill in each of these areas (see Table 1). All post-intervention differences obtained Hedges' *g* values that reflect medium to large effect sizes (0.58–0.80).

### Difficulties in Emotional Regulation

For both the *Impulse* and *Clarity* subscales of the DERS-SF and the total score, the mean post-intervention scores for the comparison group remained essentially unchanged from the pre-intervention scores. For students in the intervention group, the mean scores decreased significantly from pre- to post-intervention for both subscales and the total score. For this group, results indicated a statistically significant change from pre- to post-intervention after controlling for pre-intervention levels for the *Clarity* subscale, the *Impulse* subscale, and the total score. Lower scores on the DERS-SF reflected less difficulty with emotional regulation (see Table 1). Post-intervention differences obtained Hedges’ *g* values that reflected a medium effect size for the *Clarity* subscale (0.75) and large effect sizes for the *Impulse* subscale (1.50) and the total score (1.31).

### Resilience

For the total Resilience scale and each of the RSCA subscales, the mean post-intervention scores for the comparison group were slightly lower than the pre-intervention scores. For the intervention group, the mean scores on all scales increased significantly from pre-to post-intervention. The change from pre- to post- after controlling for pre-intervention levels was significant for the Total Resilience scale, the *Sense of Relatedness* subscale, the *Sense of Mastery* subscale, and the *Optimism* subscale. Higher scores on the RSCA indicated higher levels of resilience (see Table 1). Hedge’s *g* values for the Total Resilience scale, the *Sense of Relatedness* subscale, the *Sense of Mastery* subscale, and the *Optimism* subscale ranged from 0.68 to 0.81 indicating medium to large effect sizes.

## Discussion

The SPARK Child Mentoring program is a resilience-focused school-based SEL program designed to uncover innate resilience, promote natural emotional well-being, and facilitate school success in children and youth. SPARK is a manualized program that includes age-appropriate lessons that are delivered sequentially in a group format over consecutive weeks by trained facilitators. The program is based on the three principles of how the mind functions and assumes that sufficient understanding and insights around these principles will naturally draw out children’s social and emotional competencies. The development of social and emotional competencies occurs over time as children progress through different developmental stages, navigating different developmental tasks and learning new skills. In this way, students of different ages demonstrate their social and emotional competence in ways that are dependent on their developmental level (Denham, 2018).

We employed a randomized controlled trial with pre- and post-intervention measurement. Fidelity data support that the intervention was delivered as intended and that students randomly assigned to receive the intervention received the intended dosage. Results demonstrated that students who received the intervention increased in their knowledge regarding the three principles of how the mind functions. By helping students to understand these principles, they gained valuable insights into the resources and abilities they possess within themselves. It is through these insights that students developed important social and emotional competencies that are critical to overall positive development and adaptive functioning.

Results from this study also provided evidence for the potential of the SPARK program to affect positive change in students’ emotional regulation. Emotional regulation is an important aspect of social and emotional learning that has been linked to school engagement, motivation, academic achievement, mental health, and the establishment of healthy relationships (Djambazova-Popordanoska, 2016; Kwon et al., 2017). Children often do not realize that they can use their feelings and emotional responses to challenging situations to help them gauge whether their thinking is disordered. By helping children to understand the connection between their thoughts and their emotions, they can learn to recognize when they need to quiet down and allow their innate well-being and common sense to re-surface, thus allowing them to effectively manage how they respond to their emotions.

Findings from this study also provided support for the effectiveness of the SPARK program in positively impacting students’ self-reported resilience. Children who are confident to interact with the environment, feel securely connected to individuals in a social context, and have a positive attitude about the world in general and their own lives are better positioned to deal with hardships in a healthy and productive way (Prince-Embury, 2007). This resilience has the potential to positively impact their outlook, self-assurance, self-worth, and sense of well-being (Mak et al., 2011). Because children may not have realized that the resource of their innate mental well-being and resilience is always available to them, they can easily have fallen into a pattern marked by negative developmental outcomes. The results from this study are encouraging because they demonstrated that students can learn to recognize and engage their innate resilience to help them navigate the different tasks and challenges of development. This is especially important for students from economically disadvantaged and minority backgrounds who may be exposed to multiple and recurring risk factors. Helping these students uncover their innate resilience might be an effective way to reduce educational and health disparities in children and youth.

### Limitations

There are several limitations to our study. This investigation involved a relatively small sample size recruited from only one school within a single public-school district and regional geographic area. While the study included a diverse student sample, this limits the generalizability of findings from our study and highlights the need for additional research to strengthen evidence for the effectiveness of the SPARK Child Mentoring program. An additional limitation is the relatively short duration between pre- and post-intervention assessment (i.e., 15 weeks). Future research that incorporates longer-term follow up assessment is needed to determine if intervention effects are maintained over time. Despite noted limitations, there are several strengths to this research. First, the use of a randomized design with measurement at two time points allowed for the examination of the effects of the SPARK program on multiple indicators of positive functioning. Second, high levels of program participation and fidelity provided evidence for the validity of study findings. Finally, by controlling for outcome measures at pre-test, results from this study should have yielded a more accurate evaluation of program effectiveness (Corcoran et al., 2018).

### Future Directions

As the first systematic evaluation of the SPARK Child Mentoring program, this study is an initial step toward building evidence in support of the program and its impact on elementary age students. Our study focused primarily on short-term outcomes of the SPARK program. Future investigations of program effectiveness would benefit from the evaluation of longer-term outcomes to determine the potential to prevent maladaptive outcomes for students who receive the intervention. Future research may also focus on whether positive program impacts would be evidenced if school staff were trained as facilitators to deliver the program. Finally, additional research that focuses on understanding the characteristics of the students for whom the SPARK program is most effective would be of great benefit to schools in determining which program best fits the needs of their student population.
